# Retraction: Activation of the AMP-Activated Protein Kinase (AMPK) by Nitrated Lipids in Endothelial Cells

**DOI:** 10.1371/journal.pone.0233408

**Published:** 2020-05-19

**Authors:** The PLOS ONE Editors

Following the publication of this article [1], concerns were raised regarding the western blot data reported in Figs 1, 3, 4, 5, 6 and 7:

- Lanes 1 and 4–8 of the p-AMPK panel of Fig 1E appear similar to lanes 1–6 of the p-AMPK panel of Fig 7C. The panels represent data from the same experiment.
- Lanes 1 and 4–8 of the AMPK panel of Fig 1E appear similar to lanes 1–6 of the AMPK panel of Fig 7C. The panels represent data from the same experiment.
- Lanes 1–6 of the β-actin panel of Fig 1A appear similar to lanes 1–6 of the β-actin panel of Fig 7C.
- Lane 1 of the HIF-1α lane of Fig 3A appears similar to lane 1 of the HIF-1α lane of Fig 4C. Both bands represent the same control sample. However, the appearance of similar bands in otherwise different panels suggests the control band for one of the figures may have been spliced from a separate blot.
- In the β-actin panel of Fig 3A, the following bands appear similar:
  - lanes 3, 7, and 11;
  - lanes 4 and 8;
  - lanes 5 and 9;
  - lanes 6 and 10.
- Lanes 1–6 of the β-actin panel of Fig 3A appear similar to lanes 1–6 of the β-actin panel of Fig 4C.
- In the AMPK panel of Fig 5A, the bands in lanes 6–8 appear similar to the bands in lanes 9–11 respectively.
- In the p-AMPK panel of Fig 5A, the bands in lanes 1–3 appear similar to the bands in lanes 9–11 respectively.
- In the β-actin panel of Fig 5A, the bands in lanes 6–8 appear similar to the bands in lanes 9–11 respectively.
- Lanes 2–7 of the β-actin panel of Fig 5A appear similar to lanes 1–6 of the β-actin panel of Fig 7B.
- In the p-AMPK panel of Fig 6A, the band in lane 1 appears similar to the band in lane 6.
- In the eNOS panel of Fig 6B, the bands in lanes 1 and 2 appear similar to the bands in lanes 3 and 4 respectively.
- In the p-eNOS panel of Fig 7A, the band in lane 3 appears similar to the band in lane 4.
- In the p-eNOS panel of Fig 7B, the band in lane 1 appears similar to the band in lane 2.

The authors have indicated that the underlying data for Figs 3 and 4 are unavailable. Cropped panels and replicate experiment data for Figs 1, 5, 6 and 7 have been provided, but these do not resolve the image concerns outlined.

In light of the concerns affecting multiple figure panels that question the integrity of these data, the *PLOS ONE* Editors retract this article.

PS and M-HZ agreed with the retraction. YW did not agree with the retraction. YD either did not respond directly or could not be reached.
